# 9-[3-(Dimethyl­amino)­prop­yl]-10,10-dimethyl-9,10-dihydro­anthracen-9-ol

**DOI:** 10.1107/S1600536812050088

**Published:** 2013-01-04

**Authors:** Manpreet Kaur, Ray J. Butcher, Mehmet Akkurt, H. S. Yathirajan, B. Nagaraj

**Affiliations:** a Department of Studies in Chemistry, University of Mysore, Manasagangotri, Mysore 570 006, India; b Department of Chemistry, Howard University, 525 College Street NW, Washington, DC 20059, USA; c Department of Physics, Faculty of Sciences, Erciyes University, 38039 Kayseri, Turkey; d Department of Biotechnology, Shridevi Institute of Engineering & Technology, Tumkur 572 106, India

## Abstract

The asymmetric unit of the title compound, C_21_H_27_NO, contains two mol­ecules (*A* and *B*). In mol­ecule *A*, the central ring of the anthrone unit adopts a shallow boat conformation and the dihedral angle between the benzene rings is 18.96 (7)°. In mol­ecule *B*, the central ring is close to being planar (r.m.s. deviation = 0.078 Å) and the dihedral angle between the aromatic rings is 7.82 (7)°. In the crystal, mol­ecules are linked by O—H⋯N hydrogen bonds, forming zigzag *C*(7) chains of alternating *A* and *B* mol­ecules running parallel to [100]. The structure also features weak C—H⋯O and C—H⋯π inter­actions.

## Related literature

For a historical perspective on the applications of anthrone, see: Trevelyan (1952 ▶). For related structures see: Abboud *et al.* (1991 ▶); Fun *et al.* (2010 ▶, 2011 ▶); Siddaraju *et al.* (2011 ▶)**;** Yannoni & Silverman (1966 ▶).
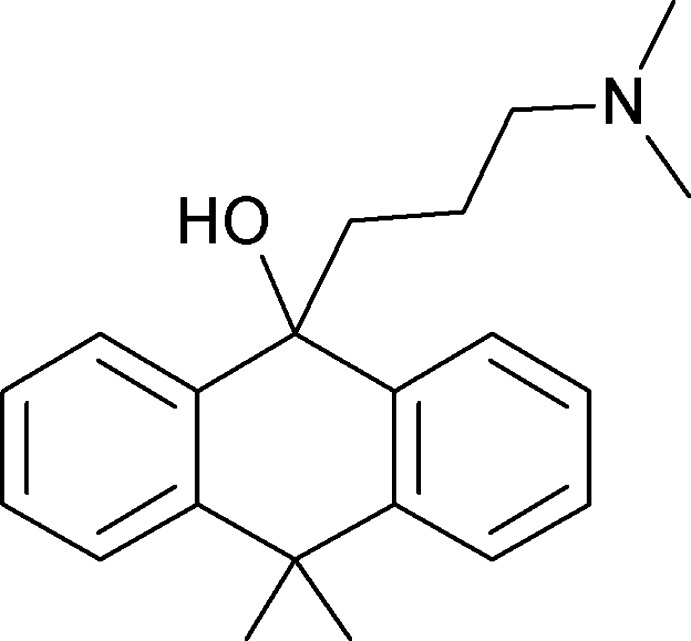


## Experimental

### 

#### Crystal data

C_21_H_27_NO
*M* *_r_* = 309.44
Monoclinic, 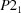

*a* = 11.79596 (9) Å
*b* = 9.17559 (7) Å
*c* = 16.75788 (13) Å
β = 92.2372 (7)°
*V* = 1812.41 (2) Å^3^
*Z* = 4
Cu *K*α radiation
μ = 0.53 mm^−1^
*T* = 123 K
0.41 × 0.34 × 0.27 mm

#### Data collection

Agilent Xcalibur (Ruby, Gemin) diffractometer
Absorption correction: multi-scan (*CrysAlis PRO*; Agilent, 2012 ▶) *T*_min_ = 0.838, *T*_max_ = 1.000
12252 measured reflections
5579 independent reflections
5539 reflections with *I* > 2σ(*I*)
*R*_int_ = 0.019

#### Refinement

*R*[*F* ^2^ > 2σ(*F* ^2^)] = 0.034
*wR*(*F* ^2^) = 0.091
*S* = 1.06
5579 reflections
425 parameters
1 restraint
H-atom parameters constrained
Δρ_max_ = 0.23 e Å^−3^
Δρ_min_ = −0.23 e Å^−3^
Absolute structure: Flack (1983 ▶), 1564 Freidel pairs
Flack parameter: 0.08 (17)

### 

Data collection: *CrysAlis PRO* (Agilent, 2012 ▶); cell refinement: *CrysAlis PRO*; data reduction: *CrysAlis PRO*; program(s) used to solve structure: *SHELXS97* (Sheldrick, 2008 ▶); program(s) used to refine structure: *SHELXL97* (Sheldrick, 2008 ▶); molecular graphics: *ORTEP-3* (Farrugia, 2012 ▶); software used to prepare material for publication: *WinGX* (Farrugia, 2012 ▶) and *PLATON* (Spek, 2009 ▶).

## Supplementary Material

Crystal structure: contains datablock(s) global, I. DOI: 10.1107/S1600536812050088/hb7006sup1.cif

Structure factors: contains datablock(s) I. DOI: 10.1107/S1600536812050088/hb7006Isup2.hkl

Supplementary material file. DOI: 10.1107/S1600536812050088/hb7006Isup3.cml

Additional supplementary materials:  crystallographic information; 3D view; checkCIF report

## Figures and Tables

**Table 1:** Hydrogen-bond geometry (Å, °) *Cg*1 and *Cg*2 are the centroids of the C11*A*–C16*A* and C2*B*–C7*B* benzene rings, respectively.

*D*—H⋯*A*	*D*—H	H⋯*A*	*D*⋯*A*	*D*—H⋯*A*
O1*A*—H1*A*⋯N1*B*	0.84	2.03	2.8659 (16)	170
O1*B*—H1*B*⋯N1*A* ^i^	0.84	2.03	2.8428 (16)	161
C17*A*—H17*B*⋯O1*B* ^ii^	0.99	2.56	3.3166 (17)	133
C20*B*—H20*F*⋯*Cg*1	0.98	2.82	3.5941 (17)	136
C21*A*—H21*A*⋯*Cg*2^ii^	0.98	2.97	3.8017 (18)	143

Symmetry codes: (i) 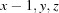; (ii) 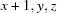.

**Fig. 1.**
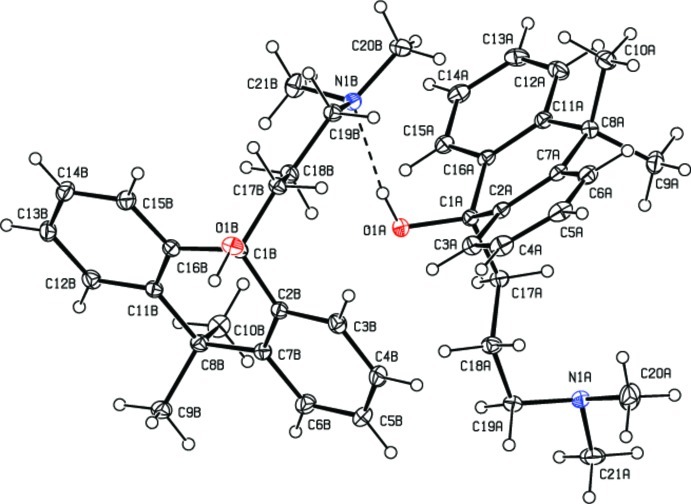
View of the the two molecules (A and B) in the asymmetric unit, with displacement ellipsoids for non-H atoms drawn at the 30% probability level.

**Fig. 2.**
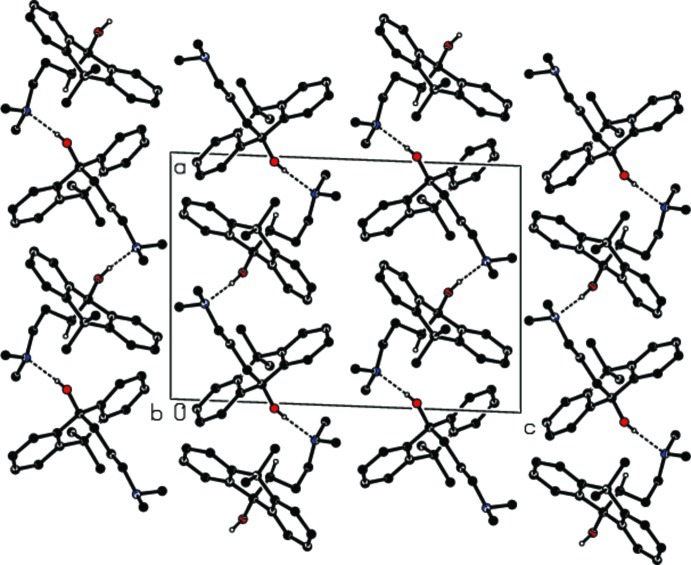
Crystal packing of the title compound, showing the O—H···N hydrogen bonding chains (dashed lines). H atoms not involved in the hydrogen bond interactions are omitted for clarity.

## supplementary crystallographic information

### Comment

Anthracene and its derivatives are long known polycyclic aromatic compounds showing a high potential for use in materials science (*e.g.* fluorescence probing, photochromic systems, electroluminescence) and several reviews have been published. Anthrone is a tricyclic aromatic hydrocarbon which is used for a popular cellulose assay and in the colorometric determination of carbohydrates (Trevelyan, 1952) and anthracene itself is used in the production of red dye alizarin. The crystal structures of 9,9,10,10-tetrachloro-9,10-dihydroanthracene (Yannoni & Silverman, 1966), *cis*-9,10-dibenzyl-9,10-dihydroanthracene (Abboud *et al.*, 1991), 9,9-dimethyl-9,10-dihydroanthracene (Siddaraju *et al.*, 2011); 10,10-dimethylanthrone (Fun *et al.*, 2010) and melitracenium chloride (Fun *et al.*, 2011) have been reported.

As part of our studies in this area, this paper reports the crystal structure of the title compound (I).

As shown in Fig. 1, there are two crystallographically independent molecules (A with the suffix A and B with the suffix B) in the asymmetric unit. In molecule A, the cyclohexane ring (C1A/C2A/C7A/C8A/C11A/C16A) adopts a shallow boat conformation, while the anthracene unit (C1B–C8B/C11B–C16B) with the cyclohexane ring in molecule B, is nearly planar, with a maximum deviation of 0.216 (1) Å for C1B. In molecules A and B, the dihedral angles between the terminal benzene rings are 18.96 (7) and 7.82 (7)°, respectively. In both molecules A and B, the values of the bond lengths and angles agree with each other.

The crystal structure is stabilized by O—H···N hydrogen bonds, forming zigzag C(7) chains running parallel to the [100] direction (Table 1, Fig. 2). Further stabilization is provided by C—H···π interactions (Table 1), involving the C11A–C16A (centroid *Cg*1) and C2B–C7B (centroid *Cg*2) benzene rings.

### Experimental

The title compound was obtained as a gift sample from *R. L*. Fine Chem, Bengaluru, India. Colourless prisms were obtained from toluene solution by slow evaporation (m.p.: 395 – 397 K).

### Refinement

All H atoms were positioned geometrically and refined using the riding-model approximation [O—H = 0.84 Å, aromatic C—H = 0.95 Å, methylene C—H = 0.99 Å and methyl C—H = 0.98 Å, and with *U*_iso_(H) = 1.2 or 1.5 *U*_eq_(parent atom)].

### Crystal data

**Table:**

C_21_H_27_NO	*F*(000) = 672
*M**_r_* = 309.44	*D*_x_ = 1.134 Mg m^−^^3^
Monoclinic, *P*2_1_	Cu *K*α radiation, λ = 1.54184 Å
Hall symbol: P 2yb	Cell parameters from 10729 reflections
*a* = 11.79596 (9) Å	θ = 2.6–75.5°
*b* = 9.17559 (7) Å	µ = 0.53 mm^−^^1^
*c* = 16.75788 (13) Å	*T* = 123 K
β = 92.2372 (7)°	Prism, colourless
*V* = 1812.41 (2) Å^3^	0.41 × 0.34 × 0.27 mm
*Z* = 4	

### Data collection

**Table:**

Agilent Xcalibur (Ruby, Gemin) diffractometer	5579 independent reflections
Radiation source: Enhance (Cu) X-ray Source	5539 reflections with *I* > 2σ(*I*)
Graphite monochromator	*R*_int_ = 0.019
Detector resolution: 10.5081 pixels mm^-1^	θ_max_ = 75.6°, θ_min_ = 2.6°
ω scans	*h* = −14→14
Absorption correction: multi-scan (*CrysAlis PRO*; Agilent, 2012)	*k* = −11→8
*T*_min_ = 0.838, *T*_max_ = 1.000	*l* = −21→19
12252 measured reflections	

### Refinement

**Table:**

Refinement on *F*^2^	Secondary atom site location: difference Fourier map
Least-squares matrix: full	Hydrogen site location: inferred from neighbouring sites
*R*[*F*^2^ > 2σ(*F*^2^)] = 0.034	H-atom parameters constrained
*wR*(*F*^2^) = 0.091	*w* = 1/[σ^2^(*F*_o_^2^) + (0.0611*P*)^2^ + 0.2469*P*] where *P* = (*F*_o_^2^ + 2*F*_c_^2^)/3
*S* = 1.06	(Δ/σ)_max_ < 0.001
5579 reflections	Δρ_max_ = 0.23 e Å^−^^3^
425 parameters	Δρ_min_ = −0.23 e Å^−^^3^
1 restraint	Absolute structure: Flack (1983), 1564 Freidel pairs
Primary atom site location: structure-invariant direct methods	Flack parameter: 0.08 (17)

### Special details

**Table:**

Geometry. Bond distances, angles *etc*. have been calculated using the rounded fractional coordinates. All su's are estimated from the variances of the (full) variance-covariance matrix. The cell e.s.d.'s are taken into account in the estimation of distances, angles and torsion angles
Refinement. Refinement on *F*^2^ for ALL reflections except those flagged by the user for potential systematic errors. Weighted *R*-factors *wR* and all goodnesses of fit *S* are based on *F*^2^, conventional *R*-factors *R* are based on *F*, with *F* set to zero for negative *F*^2^. The observed criterion of *F*^2^ > σ(*F*^2^) is used only for calculating -*R*-factor-obs *etc*. and is not relevant to the choice of reflections for refinement. *R*-factors based on *F*^2^ are statistically about twice as large as those based on *F*, and *R*-factors based on ALL data will be even larger.

### Fractional atomic coordinates and isotropic or equivalent isotropic displacement parameters (Å^2^)

**Table:**

	*x*	*y*	*z*	*U*_iso_*/*U*_eq_	
O1A	0.52432 (8)	0.52299 (12)	0.20645 (6)	0.0228 (3)	
N1A	0.85652 (10)	0.40869 (15)	0.41308 (7)	0.0237 (3)	
C1A	0.60985 (11)	0.62043 (17)	0.23606 (8)	0.0193 (4)	
C2A	0.55937 (11)	0.73586 (17)	0.28963 (8)	0.0199 (4)	
C3A	0.46633 (12)	0.69720 (19)	0.33493 (8)	0.0253 (4)	
C4A	0.42360 (13)	0.7927 (2)	0.39029 (9)	0.0295 (5)	
C5A	0.47415 (14)	0.9279 (2)	0.40181 (9)	0.0293 (4)	
C6A	0.56334 (14)	0.96866 (19)	0.35539 (9)	0.0268 (4)	
C7A	0.60672 (12)	0.87498 (17)	0.29768 (8)	0.0213 (4)	
C8A	0.70315 (12)	0.92805 (18)	0.24634 (8)	0.0236 (4)	
C9A	0.81499 (13)	0.9343 (2)	0.29815 (10)	0.0322 (5)	
C10A	0.67377 (15)	1.08296 (19)	0.21445 (9)	0.0314 (5)	
C11A	0.72071 (12)	0.82803 (17)	0.17482 (8)	0.0216 (4)	
C12A	0.78771 (13)	0.87730 (19)	0.11290 (9)	0.0286 (4)	
C13A	0.80216 (14)	0.7953 (2)	0.04513 (9)	0.0299 (5)	
C14A	0.75036 (13)	0.65980 (19)	0.03742 (8)	0.0268 (4)	
C15A	0.68707 (12)	0.60713 (18)	0.09913 (8)	0.0231 (4)	
C16A	0.67240 (11)	0.68958 (17)	0.16807 (7)	0.0196 (4)	
C17A	0.69843 (12)	0.52701 (17)	0.28362 (8)	0.0212 (4)	
C18A	0.65672 (12)	0.44957 (19)	0.35742 (9)	0.0251 (4)	
C19A	0.74501 (12)	0.34399 (18)	0.39327 (8)	0.0246 (4)	
C20A	0.84879 (17)	0.5244 (2)	0.47211 (10)	0.0423 (6)	
C21A	0.93342 (14)	0.2945 (2)	0.44273 (11)	0.0380 (5)	
O1B	−0.02159 (8)	0.56177 (12)	0.29642 (6)	0.0235 (3)	
N1B	0.39162 (10)	0.69996 (15)	0.09723 (7)	0.0212 (3)	
C1B	0.07392 (11)	0.49584 (16)	0.26043 (8)	0.0196 (4)	
C2B	0.15591 (11)	0.43394 (17)	0.32421 (8)	0.0201 (4)	
C3B	0.18045 (13)	0.52023 (18)	0.39179 (8)	0.0250 (4)	
C4B	0.25606 (14)	0.4748 (2)	0.45149 (9)	0.0306 (5)	
C5B	0.31078 (15)	0.3418 (2)	0.44413 (9)	0.0327 (5)	
C6B	0.28736 (14)	0.25557 (19)	0.37784 (9)	0.0296 (4)	
C7B	0.20841 (12)	0.29867 (17)	0.31727 (8)	0.0211 (4)	
C8B	0.18437 (12)	0.19362 (17)	0.24809 (8)	0.0224 (4)	
C9B	0.15009 (15)	0.04459 (18)	0.28289 (9)	0.0300 (5)	
C10B	0.29358 (13)	0.1738 (2)	0.20098 (9)	0.0303 (4)	
C11B	0.08826 (12)	0.24672 (17)	0.19160 (8)	0.0209 (4)	
C12B	0.05074 (13)	0.15525 (18)	0.12838 (8)	0.0253 (4)	
C13B	−0.03536 (14)	0.19642 (19)	0.07467 (8)	0.0283 (4)	
C14B	−0.08846 (13)	0.3309 (2)	0.08354 (9)	0.0302 (5)	
C15B	−0.05316 (12)	0.42182 (19)	0.14530 (9)	0.0263 (4)	
C16B	0.03580 (11)	0.38222 (17)	0.19905 (8)	0.0206 (4)	
C17B	0.12953 (12)	0.62550 (17)	0.21848 (8)	0.0210 (4)	
C18B	0.23231 (12)	0.58734 (17)	0.17024 (8)	0.0221 (4)	
C19B	0.28519 (12)	0.72540 (17)	0.13739 (8)	0.0218 (4)	
C20B	0.44276 (13)	0.8400 (2)	0.07787 (10)	0.0290 (4)	
C21B	0.37443 (14)	0.6135 (2)	0.02439 (9)	0.0321 (5)	
H3AA	0.43220	0.60420	0.32750	0.0300*	
H1A	0.47910	0.56790	0.17520	0.0340*	
H4AA	0.36010	0.76570	0.42020	0.0350*	
H5AA	0.44770	0.99230	0.44140	0.0350*	
H6AA	0.59610	1.06250	0.36270	0.0320*	
H9AA	0.83590	0.83580	0.31590	0.0480*	
H9AB	0.80410	0.99640	0.34480	0.0480*	
H9AC	0.87560	0.97470	0.26650	0.0480*	
H10A	0.60740	1.07760	0.17750	0.0470*	
H10B	0.73840	1.12210	0.18640	0.0470*	
H10C	0.65700	1.14690	0.25930	0.0470*	
H12A	0.82400	0.96950	0.11780	0.0340*	
H13A	0.84740	0.83130	0.00380	0.0360*	
H14A	0.75820	0.60380	−0.00970	0.0320*	
H15A	0.65310	0.51350	0.09450	0.0280*	
H17A	0.72820	0.45260	0.24720	0.0250*	
H17B	0.76270	0.59090	0.30040	0.0250*	
H18A	0.58650	0.39510	0.34280	0.0300*	
H18B	0.63800	0.52310	0.39810	0.0300*	
H19A	0.71500	0.30140	0.44250	0.0300*	
H19B	0.75530	0.26330	0.35500	0.0300*	
H20A	0.79990	0.60250	0.45050	0.0630*	
H20B	0.92470	0.56340	0.48490	0.0630*	
H20C	0.81650	0.48520	0.52070	0.0630*	
H21A	1.00880	0.33620	0.45430	0.0570*	
H21B	0.93880	0.21810	0.40220	0.0570*	
H21C	0.90430	0.25250	0.49160	0.0570*	
H1B	−0.05440	0.49960	0.32420	0.0350*	
H3BA	0.14410	0.61210	0.39650	0.0300*	
H4BA	0.27060	0.53400	0.49730	0.0370*	
H5BA	0.36410	0.31020	0.48440	0.0390*	
H10D	0.31570	0.26760	0.17840	0.0460*	
H10E	0.27940	0.10340	0.15780	0.0460*	
H10F	0.35480	0.13780	0.23700	0.0460*	
H6BA	0.32550	0.16500	0.37310	0.0360*	
H9BA	0.08440	0.05740	0.31620	0.0450*	
H12B	0.08560	0.06270	0.12250	0.0300*	
H9BB	0.21370	0.00470	0.31530	0.0450*	
H13B	−0.05820	0.13350	0.03200	0.0340*	
H9BC	0.13050	−0.02280	0.23910	0.0450*	
H14B	−0.14840	0.35970	0.04740	0.0360*	
H15B	−0.08980	0.51310	0.15150	0.0320*	
H17C	0.07180	0.67200	0.18230	0.0250*	
H17D	0.15300	0.69830	0.25950	0.0250*	
H18C	0.20880	0.52210	0.12550	0.0260*	
H18D	0.28910	0.53520	0.20470	0.0260*	
H19C	0.23010	0.77160	0.09910	0.0260*	
H19D	0.29970	0.79470	0.18190	0.0260*	
H20D	0.45540	0.89700	0.12690	0.0430*	
H20E	0.39180	0.89360	0.04080	0.0430*	
H20F	0.51540	0.82340	0.05290	0.0430*	
H21D	0.44650	0.60420	−0.00230	0.0480*	
H21E	0.31870	0.66190	−0.01150	0.0480*	
H21F	0.34660	0.51640	0.03820	0.0480*	

### Atomic displacement parameters (Å^2^)

**Table:**

	*U*^11^	*U*^22^	*U*^33^	*U*^12^	*U*^13^	*U*^23^
O1A	0.0206 (5)	0.0237 (5)	0.0239 (5)	−0.0039 (4)	−0.0010 (4)	0.0011 (4)
N1A	0.0259 (6)	0.0257 (7)	0.0193 (5)	−0.0023 (5)	−0.0004 (4)	0.0028 (5)
C1A	0.0179 (6)	0.0216 (7)	0.0182 (6)	−0.0007 (6)	−0.0002 (5)	0.0016 (5)
C2A	0.0178 (6)	0.0248 (7)	0.0170 (6)	0.0030 (6)	−0.0017 (5)	0.0019 (5)
C3A	0.0227 (7)	0.0288 (8)	0.0244 (6)	0.0012 (6)	0.0025 (5)	0.0017 (6)
C4A	0.0246 (7)	0.0394 (10)	0.0250 (7)	0.0065 (7)	0.0063 (6)	0.0029 (7)
C5A	0.0330 (8)	0.0334 (9)	0.0216 (6)	0.0139 (7)	0.0007 (6)	−0.0018 (6)
C6A	0.0327 (7)	0.0251 (8)	0.0221 (6)	0.0046 (7)	−0.0043 (5)	−0.0016 (6)
C7A	0.0222 (6)	0.0244 (8)	0.0170 (6)	0.0029 (6)	−0.0038 (5)	0.0014 (5)
C8A	0.0254 (7)	0.0227 (7)	0.0224 (6)	−0.0037 (6)	−0.0017 (5)	0.0006 (6)
C9A	0.0282 (7)	0.0373 (10)	0.0306 (7)	−0.0078 (7)	−0.0051 (6)	−0.0024 (7)
C10A	0.0436 (9)	0.0231 (8)	0.0274 (7)	−0.0041 (7)	0.0008 (6)	0.0011 (6)
C11A	0.0198 (6)	0.0244 (8)	0.0204 (6)	0.0003 (6)	−0.0009 (5)	0.0022 (6)
C12A	0.0279 (7)	0.0269 (8)	0.0312 (7)	−0.0039 (7)	0.0049 (6)	0.0055 (7)
C13A	0.0302 (8)	0.0348 (9)	0.0252 (7)	0.0031 (7)	0.0090 (6)	0.0091 (7)
C14A	0.0286 (7)	0.0329 (9)	0.0190 (6)	0.0075 (7)	0.0024 (5)	0.0003 (6)
C15A	0.0215 (6)	0.0250 (7)	0.0226 (6)	0.0032 (6)	−0.0002 (5)	0.0000 (6)
C16A	0.0161 (6)	0.0254 (8)	0.0171 (6)	0.0022 (6)	−0.0008 (4)	0.0022 (6)
C17A	0.0194 (6)	0.0234 (7)	0.0209 (6)	0.0016 (6)	0.0018 (5)	0.0024 (6)
C18A	0.0227 (6)	0.0284 (8)	0.0243 (6)	0.0007 (6)	0.0034 (5)	0.0059 (6)
C19A	0.0272 (7)	0.0236 (7)	0.0228 (6)	−0.0031 (6)	0.0002 (5)	0.0056 (6)
C20A	0.0493 (10)	0.0491 (12)	0.0284 (8)	−0.0105 (10)	0.0013 (7)	−0.0117 (8)
C21A	0.0283 (8)	0.0422 (10)	0.0432 (9)	−0.0010 (8)	−0.0035 (7)	0.0227 (8)
O1B	0.0211 (5)	0.0223 (5)	0.0274 (5)	0.0032 (4)	0.0067 (4)	0.0035 (4)
N1B	0.0191 (5)	0.0239 (6)	0.0207 (5)	−0.0001 (5)	0.0013 (4)	0.0020 (5)
C1B	0.0181 (6)	0.0188 (7)	0.0221 (6)	0.0009 (6)	0.0031 (5)	0.0018 (5)
C2B	0.0197 (6)	0.0213 (7)	0.0194 (6)	−0.0016 (6)	0.0021 (5)	0.0005 (6)
C3B	0.0271 (7)	0.0230 (8)	0.0250 (7)	−0.0022 (6)	0.0035 (5)	−0.0028 (6)
C4B	0.0377 (8)	0.0315 (9)	0.0223 (7)	−0.0074 (7)	−0.0030 (6)	−0.0036 (6)
C5B	0.0368 (8)	0.0343 (9)	0.0260 (7)	−0.0019 (8)	−0.0110 (6)	0.0026 (7)
C6B	0.0316 (8)	0.0261 (8)	0.0306 (7)	0.0043 (7)	−0.0058 (6)	0.0008 (7)
C7B	0.0225 (6)	0.0208 (7)	0.0200 (6)	−0.0003 (6)	0.0006 (5)	0.0011 (5)
C8B	0.0252 (7)	0.0190 (7)	0.0228 (6)	0.0024 (6)	0.0002 (5)	−0.0001 (6)
C9B	0.0430 (9)	0.0195 (8)	0.0271 (7)	0.0012 (7)	−0.0040 (6)	0.0014 (6)
C10B	0.0284 (7)	0.0334 (9)	0.0293 (7)	0.0075 (7)	0.0028 (6)	−0.0037 (7)
C11B	0.0214 (6)	0.0224 (7)	0.0189 (6)	−0.0031 (6)	0.0027 (5)	0.0015 (6)
C12B	0.0302 (7)	0.0237 (8)	0.0224 (6)	−0.0039 (6)	0.0046 (5)	−0.0010 (6)
C13B	0.0318 (7)	0.0321 (9)	0.0210 (6)	−0.0116 (7)	−0.0006 (5)	−0.0018 (6)
C14B	0.0242 (7)	0.0393 (10)	0.0267 (7)	−0.0062 (7)	−0.0054 (6)	0.0053 (7)
C15B	0.0210 (6)	0.0301 (8)	0.0278 (7)	−0.0008 (6)	0.0003 (5)	0.0033 (7)
C16B	0.0186 (6)	0.0232 (8)	0.0201 (6)	−0.0030 (6)	0.0032 (5)	0.0026 (5)
C17B	0.0214 (6)	0.0175 (7)	0.0241 (6)	0.0008 (6)	0.0018 (5)	0.0015 (5)
C18B	0.0202 (6)	0.0215 (8)	0.0246 (6)	−0.0008 (6)	0.0024 (5)	0.0009 (6)
C19B	0.0214 (6)	0.0212 (7)	0.0227 (6)	0.0013 (6)	0.0013 (5)	0.0011 (6)
C20B	0.0245 (7)	0.0285 (8)	0.0341 (7)	0.0012 (7)	0.0033 (6)	0.0108 (7)
C21B	0.0298 (7)	0.0418 (10)	0.0248 (7)	0.0000 (8)	0.0040 (6)	−0.0072 (7)

### Geometric parameters (Å, º)

**Table:**

O1A—C1A	1.4228 (17)	C20A—H20C	0.9800
O1A—H1A	0.8400	C20A—H20A	0.9800
O1B—C1B	1.4325 (17)	C21A—H21C	0.9800
O1B—H1B	0.8400	C21A—H21A	0.9800
N1A—C20A	1.456 (2)	C21A—H21B	0.9800
N1A—C19A	1.4693 (19)	C1B—C16B	1.520 (2)
N1A—C21A	1.460 (2)	C1B—C17B	1.541 (2)
N1B—C20B	1.461 (2)	C1B—C2B	1.5230 (19)
N1B—C19B	1.4661 (18)	C2B—C7B	1.394 (2)
N1B—C21B	1.463 (2)	C2B—C3B	1.403 (2)
C1A—C2A	1.525 (2)	C3B—C4B	1.379 (2)
C1A—C16A	1.5201 (19)	C4B—C5B	1.388 (3)
C1A—C17A	1.548 (2)	C5B—C6B	1.383 (2)
C2A—C7A	1.398 (2)	C6B—C7B	1.407 (2)
C2A—C3A	1.4042 (19)	C7B—C8B	1.526 (2)
C3A—C4A	1.385 (2)	C8B—C9B	1.547 (2)
C4A—C5A	1.387 (3)	C8B—C11B	1.528 (2)
C5A—C6A	1.384 (2)	C8B—C10B	1.548 (2)
C6A—C7A	1.405 (2)	C11B—C12B	1.409 (2)
C7A—C8A	1.532 (2)	C11B—C16B	1.397 (2)
C8A—C10A	1.553 (2)	C12B—C13B	1.383 (2)
C8A—C11A	1.530 (2)	C13B—C14B	1.394 (2)
C8A—C9A	1.552 (2)	C14B—C15B	1.381 (2)
C11A—C16A	1.395 (2)	C15B—C16B	1.404 (2)
C11A—C12A	1.404 (2)	C17B—C18B	1.524 (2)
C12A—C13A	1.378 (2)	C18B—C19B	1.524 (2)
C13A—C14A	1.389 (2)	C3B—H3BA	0.9500
C14A—C15A	1.386 (2)	C4B—H4BA	0.9500
C15A—C16A	1.3974 (19)	C5B—H5BA	0.9500
C17A—C18A	1.525 (2)	C6B—H6BA	0.9500
C18A—C19A	1.528 (2)	C9B—H9BA	0.9800
C3A—H3AA	0.9500	C9B—H9BB	0.9800
C4A—H4AA	0.9500	C9B—H9BC	0.9800
C5A—H5AA	0.9500	C10B—H10D	0.9800
C6A—H6AA	0.9500	C10B—H10E	0.9800
C9A—H9AA	0.9800	C10B—H10F	0.9800
C9A—H9AC	0.9800	C12B—H12B	0.9500
C9A—H9AB	0.9800	C13B—H13B	0.9500
C10A—H10C	0.9800	C14B—H14B	0.9500
C10A—H10B	0.9800	C15B—H15B	0.9500
C10A—H10A	0.9800	C17B—H17C	0.9900
C12A—H12A	0.9500	C17B—H17D	0.9900
C13A—H13A	0.9500	C18B—H18C	0.9900
C14A—H14A	0.9500	C18B—H18D	0.9900
C15A—H15A	0.9500	C19B—H19C	0.9900
C17A—H17A	0.9900	C19B—H19D	0.9900
C17A—H17B	0.9900	C20B—H20D	0.9800
C18A—H18B	0.9900	C20B—H20E	0.9800
C18A—H18A	0.9900	C20B—H20F	0.9800
C19A—H19B	0.9900	C21B—H21D	0.9800
C19A—H19A	0.9900	C21B—H21E	0.9800
C20A—H20B	0.9800	C21B—H21F	0.9800
			
C1A—O1A—H1A	109.00	H21A—C21A—H21B	109.00
C1B—O1B—H1B	109.00	H21A—C21A—H21C	109.00
C19A—N1A—C21A	109.01 (13)	N1A—C21A—H21B	109.00
C19A—N1A—C20A	111.64 (12)	N1A—C21A—H21A	110.00
C20A—N1A—C21A	110.27 (13)	O1B—C1B—C2B	110.51 (11)
C20B—N1B—C21B	109.66 (12)	O1B—C1B—C17B	102.81 (11)
C19B—N1B—C20B	109.28 (12)	C2B—C1B—C16B	112.46 (12)
C19B—N1B—C21B	112.09 (12)	O1B—C1B—C16B	110.96 (11)
C16A—C1A—C17A	106.22 (11)	C16B—C1B—C17B	109.92 (11)
C2A—C1A—C16A	111.32 (12)	C2B—C1B—C17B	109.76 (11)
O1A—C1A—C16A	111.05 (11)	C1B—C2B—C7B	123.06 (12)
O1A—C1A—C2A	110.73 (11)	C3B—C2B—C7B	119.45 (13)
O1A—C1A—C17A	106.66 (12)	C1B—C2B—C3B	117.48 (13)
C2A—C1A—C17A	110.66 (11)	C2B—C3B—C4B	121.50 (15)
C3A—C2A—C7A	119.76 (13)	C3B—C4B—C5B	119.42 (15)
C1A—C2A—C3A	118.32 (13)	C4B—C5B—C6B	119.69 (15)
C1A—C2A—C7A	121.84 (12)	C5B—C6B—C7B	121.63 (16)
C2A—C3A—C4A	120.98 (15)	C2B—C7B—C8B	123.77 (12)
C3A—C4A—C5A	119.57 (14)	C6B—C7B—C8B	117.95 (14)
C4A—C5A—C6A	119.71 (15)	C2B—C7B—C6B	118.28 (13)
C5A—C6A—C7A	121.78 (16)	C7B—C8B—C9B	108.40 (11)
C6A—C7A—C8A	119.36 (14)	C7B—C8B—C11B	112.38 (12)
C2A—C7A—C8A	122.59 (13)	C9B—C8B—C10B	108.88 (13)
C2A—C7A—C6A	118.05 (13)	C7B—C8B—C10B	109.15 (12)
C7A—C8A—C10A	108.88 (12)	C10B—C8B—C11B	109.49 (11)
C7A—C8A—C11A	112.05 (12)	C9B—C8B—C11B	108.48 (12)
C7A—C8A—C9A	109.34 (11)	C8B—C11B—C16B	123.31 (13)
C9A—C8A—C10A	109.40 (13)	C12B—C11B—C16B	118.04 (13)
C10A—C8A—C11A	108.34 (11)	C8B—C11B—C12B	118.65 (13)
C9A—C8A—C11A	108.80 (12)	C11B—C12B—C13B	121.85 (15)
C12A—C11A—C16A	118.26 (13)	C12B—C13B—C14B	119.59 (14)
C8A—C11A—C16A	122.84 (12)	C13B—C14B—C15B	119.40 (14)
C8A—C11A—C12A	118.90 (14)	C14B—C15B—C16B	121.37 (15)
C11A—C12A—C13A	121.64 (15)	C1B—C16B—C15B	116.78 (13)
C12A—C13A—C14A	119.81 (15)	C11B—C16B—C15B	119.72 (13)
C13A—C14A—C15A	119.38 (14)	C1B—C16B—C11B	123.41 (12)
C14A—C15A—C16A	121.05 (15)	C1B—C17B—C18B	115.22 (12)
C1A—C16A—C15A	118.24 (13)	C17B—C18B—C19B	110.23 (12)
C1A—C16A—C11A	121.87 (12)	N1B—C19B—C18B	113.78 (12)
C11A—C16A—C15A	119.78 (12)	C2B—C3B—H3BA	119.00
C1A—C17A—C18A	116.30 (12)	C4B—C3B—H3BA	119.00
C17A—C18A—C19A	112.20 (12)	C3B—C4B—H4BA	120.00
N1A—C19A—C18A	115.03 (13)	C5B—C4B—H4BA	120.00
C4A—C3A—H3AA	120.00	C4B—C5B—H5BA	120.00
C2A—C3A—H3AA	119.00	C6B—C5B—H5BA	120.00
C3A—C4A—H4AA	120.00	C5B—C6B—H6BA	119.00
C5A—C4A—H4AA	120.00	C7B—C6B—H6BA	119.00
C6A—C5A—H5AA	120.00	C8B—C9B—H9BA	109.00
C4A—C5A—H5AA	120.00	C8B—C9B—H9BB	109.00
C5A—C6A—H6AA	119.00	C8B—C9B—H9BC	109.00
C7A—C6A—H6AA	119.00	H9BA—C9B—H9BB	110.00
H9AA—C9A—H9AC	109.00	H9BA—C9B—H9BC	109.00
H9AB—C9A—H9AC	109.00	H9BB—C9B—H9BC	109.00
C8A—C9A—H9AB	109.00	C8B—C10B—H10D	109.00
C8A—C9A—H9AA	110.00	C8B—C10B—H10E	109.00
H9AA—C9A—H9AB	109.00	C8B—C10B—H10F	109.00
C8A—C9A—H9AC	109.00	H10D—C10B—H10E	110.00
C8A—C10A—H10A	109.00	H10D—C10B—H10F	109.00
C8A—C10A—H10C	109.00	H10E—C10B—H10F	109.00
C8A—C10A—H10B	109.00	C11B—C12B—H12B	119.00
H10A—C10A—H10B	109.00	C13B—C12B—H12B	119.00
H10A—C10A—H10C	110.00	C12B—C13B—H13B	120.00
H10B—C10A—H10C	109.00	C14B—C13B—H13B	120.00
C13A—C12A—H12A	119.00	C13B—C14B—H14B	120.00
C11A—C12A—H12A	119.00	C15B—C14B—H14B	120.00
C12A—C13A—H13A	120.00	C14B—C15B—H15B	119.00
C14A—C13A—H13A	120.00	C16B—C15B—H15B	119.00
C15A—C14A—H14A	120.00	C1B—C17B—H17C	108.00
C13A—C14A—H14A	120.00	C1B—C17B—H17D	108.00
C14A—C15A—H15A	119.00	C18B—C17B—H17C	108.00
C16A—C15A—H15A	119.00	C18B—C17B—H17D	108.00
C1A—C17A—H17B	108.00	H17C—C17B—H17D	108.00
H17A—C17A—H17B	107.00	C17B—C18B—H18C	110.00
C18A—C17A—H17B	108.00	C17B—C18B—H18D	110.00
C1A—C17A—H17A	108.00	C19B—C18B—H18C	110.00
C18A—C17A—H17A	108.00	C19B—C18B—H18D	110.00
C19A—C18A—H18B	109.00	H18C—C18B—H18D	108.00
C17A—C18A—H18B	109.00	N1B—C19B—H19C	109.00
C19A—C18A—H18A	109.00	N1B—C19B—H19D	109.00
H18A—C18A—H18B	108.00	C18B—C19B—H19C	109.00
C17A—C18A—H18A	109.00	C18B—C19B—H19D	109.00
N1A—C19A—H19B	108.00	H19C—C19B—H19D	108.00
H19A—C19A—H19B	108.00	N1B—C20B—H20D	109.00
N1A—C19A—H19A	108.00	N1B—C20B—H20E	109.00
C18A—C19A—H19A	108.00	N1B—C20B—H20F	109.00
C18A—C19A—H19B	109.00	H20D—C20B—H20E	109.00
N1A—C20A—H20B	109.00	H20D—C20B—H20F	110.00
N1A—C20A—H20C	109.00	H20E—C20B—H20F	109.00
H20A—C20A—H20B	109.00	N1B—C21B—H21D	109.00
N1A—C20A—H20A	109.00	N1B—C21B—H21E	110.00
H20B—C20A—H20C	109.00	N1B—C21B—H21F	109.00
H20A—C20A—H20C	109.00	H21D—C21B—H21E	109.00
N1A—C21A—H21C	109.00	H21D—C21B—H21F	109.00
H21B—C21A—H21C	109.00	H21E—C21B—H21F	110.00
			
C20A—N1A—C19A—C18A	60.80 (16)	C1A—C17A—C18A—C19A	171.98 (13)
C21A—N1A—C19A—C18A	−177.13 (12)	C17A—C18A—C19A—N1A	55.16 (17)
C21B—N1B—C19B—C18B	65.90 (15)	O1B—C1B—C2B—C3B	−43.87 (17)
C20B—N1B—C19B—C18B	−172.32 (12)	O1B—C1B—C2B—C7B	137.56 (13)
O1A—C1A—C2A—C7A	−151.17 (13)	C16B—C1B—C2B—C3B	−168.48 (12)
O1A—C1A—C2A—C3A	32.20 (17)	C16B—C1B—C2B—C7B	12.95 (18)
C17A—C1A—C2A—C3A	−85.86 (15)	C17B—C1B—C2B—C3B	68.83 (16)
C16A—C1A—C2A—C3A	156.26 (12)	C17B—C1B—C2B—C7B	−109.74 (15)
C16A—C1A—C2A—C7A	−27.11 (17)	O1B—C1B—C16B—C11B	−137.55 (13)
O1A—C1A—C16A—C15A	−33.01 (17)	O1B—C1B—C16B—C15B	45.83 (17)
C2A—C1A—C16A—C11A	27.04 (17)	C2B—C1B—C16B—C11B	−13.19 (18)
C2A—C1A—C16A—C15A	−156.89 (12)	C2B—C1B—C16B—C15B	170.19 (12)
C17A—C1A—C16A—C11A	−93.49 (16)	C17B—C1B—C16B—C11B	109.41 (15)
C17A—C1A—C16A—C15A	82.58 (15)	C17B—C1B—C16B—C15B	−67.21 (15)
O1A—C1A—C17A—C18A	−63.45 (15)	O1B—C1B—C17B—C18B	−176.96 (11)
C2A—C1A—C17A—C18A	57.07 (17)	C2B—C1B—C17B—C18B	65.43 (15)
C16A—C1A—C17A—C18A	178.02 (12)	C16B—C1B—C17B—C18B	−58.75 (15)
O1A—C1A—C16A—C11A	150.92 (13)	C1B—C2B—C3B—C4B	−178.19 (14)
C17A—C1A—C2A—C7A	90.77 (16)	C7B—C2B—C3B—C4B	0.4 (2)
C7A—C2A—C3A—C4A	−2.9 (2)	C1B—C2B—C7B—C6B	176.64 (13)
C1A—C2A—C3A—C4A	173.82 (13)	C1B—C2B—C7B—C8B	−4.0 (2)
C3A—C2A—C7A—C6A	4.1 (2)	C3B—C2B—C7B—C6B	−1.9 (2)
C1A—C2A—C7A—C6A	−172.48 (13)	C3B—C2B—C7B—C8B	177.45 (13)
C1A—C2A—C7A—C8A	7.5 (2)	C2B—C3B—C4B—C5B	1.1 (2)
C3A—C2A—C7A—C8A	−175.92 (13)	C3B—C4B—C5B—C6B	−1.1 (3)
C2A—C3A—C4A—C5A	−0.7 (2)	C4B—C5B—C6B—C7B	−0.4 (3)
C3A—C4A—C5A—C6A	3.0 (2)	C5B—C6B—C7B—C2B	1.9 (2)
C4A—C5A—C6A—C7A	−1.7 (2)	C5B—C6B—C7B—C8B	−177.48 (15)
C5A—C6A—C7A—C8A	178.12 (14)	C2B—C7B—C8B—C9B	−125.39 (15)
C5A—C6A—C7A—C2A	−1.9 (2)	C2B—C7B—C8B—C10B	116.16 (15)
C2A—C7A—C8A—C11A	13.35 (19)	C2B—C7B—C8B—C11B	−5.51 (19)
C2A—C7A—C8A—C9A	−107.36 (16)	C6B—C7B—C8B—C9B	53.97 (17)
C2A—C7A—C8A—C10A	133.17 (14)	C6B—C7B—C8B—C10B	−64.48 (17)
C6A—C7A—C8A—C11A	−166.67 (13)	C6B—C7B—C8B—C11B	173.85 (13)
C6A—C7A—C8A—C9A	72.63 (18)	C7B—C8B—C11B—C12B	−175.11 (13)
C6A—C7A—C8A—C10A	−46.85 (17)	C7B—C8B—C11B—C16B	5.30 (19)
C7A—C8A—C11A—C12A	166.18 (13)	C9B—C8B—C11B—C12B	−55.28 (17)
C9A—C8A—C11A—C16A	107.55 (16)	C9B—C8B—C11B—C16B	125.13 (15)
C7A—C8A—C11A—C16A	−13.47 (19)	C10B—C8B—C11B—C12B	63.42 (18)
C9A—C8A—C11A—C12A	−72.81 (17)	C10B—C8B—C11B—C16B	−116.18 (15)
C10A—C8A—C11A—C16A	−133.60 (14)	C8B—C11B—C12B—C13B	−179.62 (14)
C10A—C8A—C11A—C12A	46.04 (18)	C16B—C11B—C12B—C13B	0.0 (2)
C16A—C11A—C12A—C13A	2.9 (2)	C8B—C11B—C16B—C1B	4.4 (2)
C8A—C11A—C16A—C1A	−7.4 (2)	C8B—C11B—C16B—C15B	−179.04 (13)
C12A—C11A—C16A—C1A	172.99 (13)	C12B—C11B—C16B—C1B	−175.16 (13)
C12A—C11A—C16A—C15A	−3.0 (2)	C12B—C11B—C16B—C15B	1.4 (2)
C8A—C11A—C16A—C15A	176.62 (13)	C11B—C12B—C13B—C14B	−1.1 (2)
C8A—C11A—C12A—C13A	−176.77 (14)	C12B—C13B—C14B—C15B	0.9 (2)
C11A—C12A—C13A—C14A	−0.5 (2)	C13B—C14B—C15B—C16B	0.5 (2)
C12A—C13A—C14A—C15A	−1.7 (2)	C14B—C15B—C16B—C1B	175.10 (13)
C13A—C14A—C15A—C16A	1.5 (2)	C14B—C15B—C16B—C11B	−1.7 (2)
C14A—C15A—C16A—C1A	−175.27 (13)	C1B—C17B—C18B—C19B	−175.46 (11)
C14A—C15A—C16A—C11A	0.9 (2)	C17B—C18B—C19B—N1B	173.94 (11)

### Hydrogen-bond geometry (Å, º)

Cg1 and Cg2 are the centroids of the C11A–C16A and C2B–C7B benzene rings, respectively.

**Table:**

*D*—H···*A*	*D*—H	H···*A*	*D*···*A*	*D*—H···*A*
O1*A*—H1*A*···N1*B*	0.84	2.03	2.8659 (16)	170
O1*B*—H1*B*···N1*A*^i^	0.84	2.03	2.8428 (16)	161
C17*A*—H17*B*···O1*B*^ii^	0.99	2.56	3.3166 (17)	133
C20*B*—H20*F*···*Cg*1	0.98	2.82	3.5941 (17)	136
C21*A*—H21*A*···*Cg*2^ii^	0.98	2.97	3.8017 (18)	143

Symmetry codes: (i) *x*−1, *y*, *z*; (ii) *x*+1, *y*, *z*.
